# Opportunities and challenges for assigning cofactors in cryo-EM density maps of chlorophyll-containing proteins

**DOI:** 10.1038/s42003-020-01139-1

**Published:** 2020-07-30

**Authors:** Christopher J. Gisriel, Jimin Wang, Gary W. Brudvig, Donald A. Bryant

**Affiliations:** 1 Department of Chemistry, Yale University, New Haven, CT 06520 USA; 2 Department of Molecular Biophysics and Biochemistry, Yale University, New Haven, CT 06520 USA; 3 Department of Biochemistry and Molecular Biology, The Pennsylvania State University, University Park, PA 16802 USA; 4 Department of Chemistry and Biochemistry, Montana State University, Bozeman, MT 59717 USA

**Keywords:** Cryoelectron microscopy, Bacteria, Photosystem I

## Abstract

The accurate assignment of cofactors in cryo-electron microscopy maps is crucial in determining protein function. This is particularly true for chlorophylls (Chls), for which small structural differences lead to important functional differences. Recent cryo-electron microscopy structures of Chl-containing protein complexes exemplify the difficulties in distinguishing Chl *b* and Chl *f* from Chl *a*. We use these structures as examples to discuss general issues arising from local resolution differences, properties of electrostatic potential maps, and the chemical environment which must be considered to make accurate assignments. We offer suggestions for how to improve the reliability of such assignments.

## Abstract (web summary)

In this Perspective, Christopher Gisriel et al. discuss the challenges in accurate assignment of co-factors in cryo-EM, particularly for chlorophylls. They explore the factors that lead to misassignment and offer suggestions for improving reliability of cryo-EM-based assignments.

## Introduction

Cryo-electron microscopy (EM) has opened new opportunities for structural biology, particularly for photosynthesis research, because crystallization of large, multisubunit, and membrane-associated proteins is often a bottleneck for structural studies by X-ray crystallography. Since the popularization of cryo-EM following the “resolution revolution^1^,” cryo-EM structures of protein complexes involved in oxygenic photosynthesis research have begun to flood the literature^2–25^. Distinct from X-ray crystallography, which yields three-dimensional maps of electron density, cryo-EM yields three-dimensional maps that display the variation of the electrostatic potential (ESP) of molecules to which both nuclei and electrons contribute (see Supplementary Text 1). It is important to note that atoms having net negative charges contribute less to ESP maps than neutral atoms^26^. Thus, commonly occurring oxygen atoms with a partial negative charge can be particularly more challenging to identify in ESP maps obtained from cryo-EM than in electron density maps obtained by X-ray crystallography. Practically, this means that much higher resolution is required to distinguish a formyl group (chlorophyll (Chl) *b* and Chl *f*) from a methyl group (Chl *a*) in an ESP map than in an electron density map. Recent structural studies of various Chl-containing proteins nicely demonstrate the practical consequences of these issues. Nonetheless, it may still be possible to make accurate assignments of specific Chls and we offer suggestions for how to improve the reliability of these assignments.

Chls are a major subfamily of the larger class of biomolecules known as tetrapyrroles and there are approximately 15 major types of Chls and bacteriochlorophylls^27^. The structural differences among the four major types of Chls (*a*, *b*, *d*, and *f*) found in cyanobacteria and higher plants are subtle but are functionally important (Supplementary Fig. 1). Chls *b* and *f* are isomers, but the former is specialized for harvesting blue light (*λ* = 400–500 nm), while the latter is specialized for harvesting far-red light (FRL; *λ* = 700–800 nm). Accordingly, Chl *b* is found in peripheral antenna complexes, from which downhill energy transfer occurs toward the reaction center cores. The locations of Chl *f* were unclear until recently and a thorough analysis is provided below. Compared with Chl *a*, Chl *b* has a formyl group at C7, whereas Chl *f* has a formyl group at C2; Chl *a* has methyl groups at both of these positions. Chl *d* also has a formyl group at position C3 where Chl *a* has a vinyl group. Finally, divinyl-Chl *a* and divinyl-Chl *b* are minor Chls similar to Chls *a* and *b*, but they have a vinyl group at C8 instead of an ethyl moiety^27^, which improves the blue-light-harvesting capabilities of some marine *Prochlorococcus* spp. by allowing them to harvest light not absorbed by Chl *a*. The C7 formyl moiety of Chl *b* is typically found accepting an H-bond from the polypeptide or water, which is thought to confer discrimination against Chl *a* for specific recognition of Chl *b* by apo-proteins^28^ (see below), as is likely the case for Chl *f* and *d* as well. As Chls *d* and *f* have been suggested to play roles in electron transfer as well as acting as antenna pigments for FRL absorption in Photosystem I (PSI) and Photosystem II (PSII)^29,30^, proteins containing these specialized Chls have been targeted by structural biologists to gain fresh insights in subtle aspects of excitation energy transfer and energy trapping in oxygenic photosynthesis^2,3^. Whereas many of these structures are informed by previously determined X-ray crystal structures and their Chls are assigned based on analogy, some recent cryo-EM structures do not have this prior knowledge^2,3^ and the Chl assignments must be informed instead by direct observation, chemical environment, or energetic constraints, the former of which is a challenge for cryo-EM.

In this perspective, the ambiguity in Chl *b* and *f* assignments in cryo-EM structures found in the Protein Data Bank^31,32^ for light-harvesting complexes I (LHCI) and II (LHCII) of plants and algae, and FRL-acclimated PSI (FRL-PSI) from cyanobacteria are discussed. These structures are used to: (i) highlight the characteristics of ESP maps that should be considered during modeling; (ii) discuss modeling discrepancies in the current literature; (iii) propose reasonable chemical environments for Chls that exhibit formyl substituents; and (iv) suggest a strategy to quantify the assignment of unique and functionally important Chl types in ESP maps of photosynthetic macromolecules.

## Challenges of assigning (partially) negatively charged substituents in cryo-EM maps

The goal of structural biology is to obtain accurate information about the structures of biomolecules that confer their functions. Presently, cryo-EM structures of ~2.5- to 3.5 Å resolution are routinely being determined and described in publications that elucidate important molecular interactions of amino acids, nucleic acids, and the cofactors they coordinate. These structures present an opportunity to examine subtle structural differences that give rise to important biological mechanisms and they illustrate the challenges that accompany accurate assignment of structurally minor features, especially those with partial negative charges—but with critical functional significance. A common approach for identifying small differences between structures is the comparison of two isomorphous data sets. Importantly, two electron density maps obtained by X-ray crystallography can be placed on the same scale so that the maps can be directly compared to identify differences that can be used to reveal missing features. Given the fact that the same scaling cannot be done for ESP maps in cryo-EM, the next best approach is to scale the relative variance of ESP maps, as has only recently appeared in the literature for a protein involved in photosynthesis^3^. However, this approach cannot remove local resolution differences and may be problematic for resolving subtle structural differences as described below.

Providing solutions to these problems first requires an understanding of the differences between cryo-EM-derived ESP maps and X-ray crystallography-derived electron density maps, and that it is important to consider the difference between the two types of radiation used in these techniques. In the simplest case of a proton (H atom without an electron), differences between electron scattering and X-ray scattering are striking. In an electron beam, the proton has a positive scattering length that is infinite when the scattering angle is zero, but in an X-ray beam, it does not scatter at all. For neutral atoms in an X-ray beam, the forward scattering lengths are proportional to atomic number but are far less regular than those in an electron beam. For example, the forward scattering length of O in an electron beam is less than that for C. Experimental observations in cryo-EM maps abundantly confirm these theoretical expectations^33,34^.

The difference between the two kinds of radiation is even more complicated for charged atoms than neutral atoms. For cations, the differences can be conceptually viewed as a linear combination of protons and neutral atoms. For example, Mg^2+^ scatters electrons much more strongly than a neutral Mg atom in an electron beam, but they scatter similarly in an X-ray beam. Negative scattering lengths predominate for anions in ESP maps at low resolution. Without data of sufficiently high resolution, anions can have overall negative ESP values as demonstrated by carboxylate side chains^35,36^. At a distance three times the van der Waals radius of the corresponding neutral atom, the distribution of extra valence electrons can be approximated as point-charges centered at the nucleus, and their long-range ESP follows Eqn (1) (Supplementary Text 1). Within the van der Waals radius, this approximation breaks down. Nevertheless, electron scattering factors of ions can be parameterized using a van der Waals component and a Coulomb component^34,37^.

**Fig. 1:**
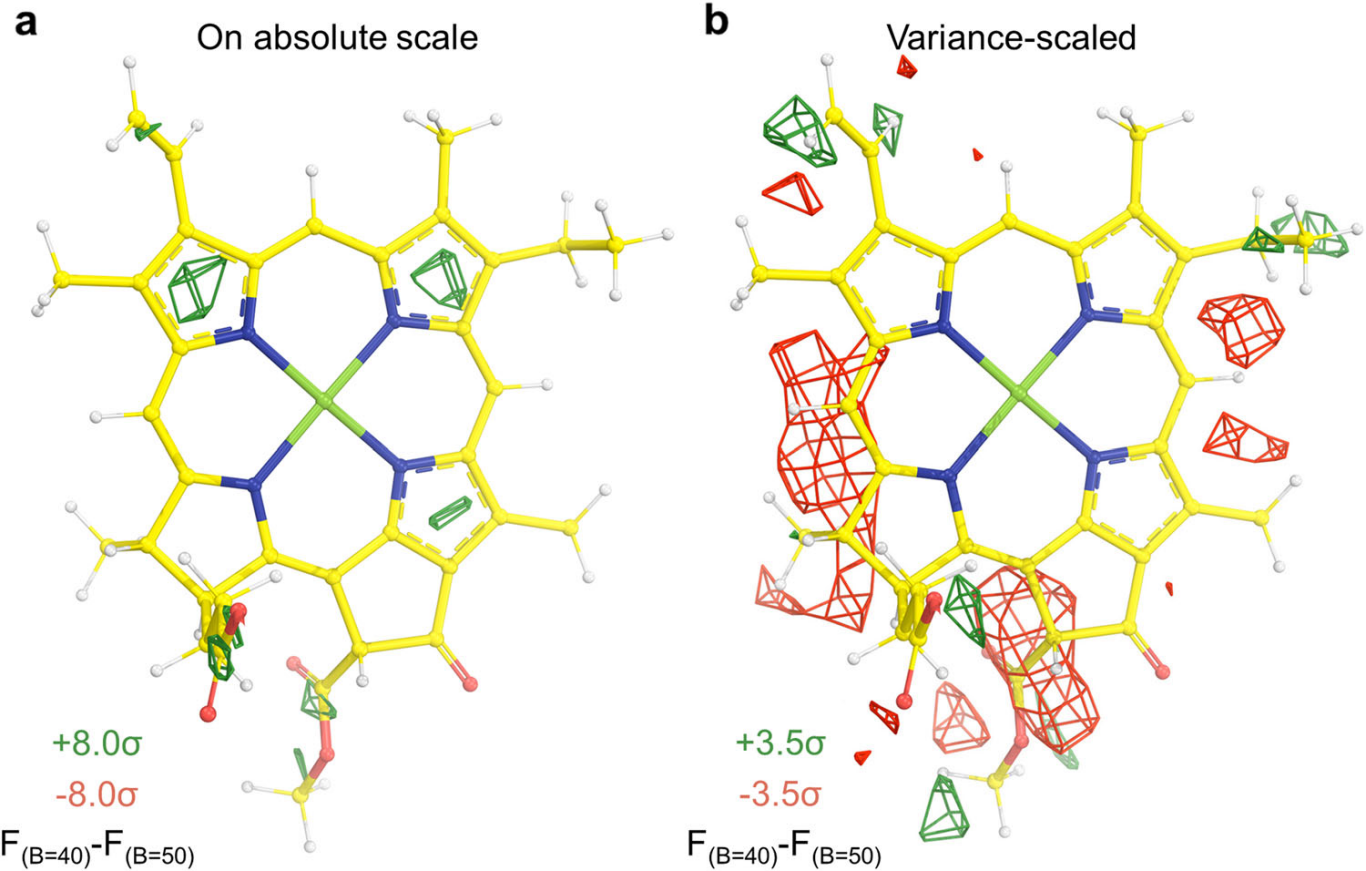
Real-space difference features between calculated Chl maps of different B-factors at 2.4 Å resolution. **a** Difference map between two calculated Chl *a* molecules with B-factors of 40 Å^2^ and 50 Å^2^ on an absolute scale without linear rescaling. **b** Difference map between two calculated Chl *a* molecules with B-factors of 40 Å^2^ and 50 Å^2^ after linear rescaling by making their variance the same without correcting for their B-factor difference. In either case, positive and negative peaks appear throughout the Fourier difference map. The size of the box used in this calculation is 25 Å × 25 Å × 25 Å.

Cryo-EM measures averaged ESP maps from a large number of single molecules at different time points of dynamic trajectory, which can be described in real space using the equilibrium locations of individual atoms and the variance of their dynamic distributions (or dynamic motions) in the form of their convolution. In reciprocal space, this convolution becomes a multiplication of the two corresponding Fourier transforms. As the ESP has both positive and negative signs, and electron density has only positive values, the interpretation of difference Fourier features also differ between X-ray-derived and cryo-EM-derived density maps. This is important in distinguishing formyl vs. methyl substituents considered here: positive differences in electric potential features cannot always be interpreted as additional atoms, nor can negative electric potential features always be interpreted as missing atoms, unless local resolution differences have been corrected, which is difficult to achieve with ESP maps. To demonstrate this point, we placed a Chl *a* model in the center of a 25 Å^3^ box and calculated one ESP map with a B-factor of 40 Å^2^ and the other with a B-factor of 50 Å^2^ using the procedure described by Wang^33^. The real-space difference was calculated after the two maps were placed on an absolute scale without any rescaling (Fig. 1a) and after linear rescaling by making their variance the same without correcting for their B-factor difference (Fig. 1b). Spurious peaks appear in many locations even though the underlying structures are identical. This problem is solved in X-ray crystallography^38^ by first scaling the two data sets being compared using the logarithmic plot of amplitudes as reciprocal resolution squares to remove their Wilson B-factor difference or resolution difference before placing them on the absolute scale. The relationship between resolution and B-factor in X-ray crystallography is well-understood and can be used to approximate the same relationship in cryo-EM^39^. The scale of an ESP map is also influenced by box size and solvent content, which can introduce even more artifactual density in difference maps. Thus, two ESP maps are never on exactly the same scale.

The demonstration in Fig. 1 illustrates that, from a theoretical perspective, peaks may appear due to scaling discrepancies in cryo-EM-derived Chl difference maps that do not arise from structural differences. These discrepancies can result in false positive and false negative assignments, i.e., a positive feature can easily be misinterpreted as an additional oxygen atom, such as the formyl substituent of Chl *b*, *d*, or *f*. Alternatively, a negative feature can cause the formyl O atom to be invisible, which might cause a Chl molecule containing a formyl group to be misassigned as Chl *a*. In addition, Fourier series termination causes ripple effects of a certain signal above the noise. As the signals are not normalized between maps, artifactual contributions to the difference density may be observed when extreme B-factor sharpening is applied to cryo-EM maps, which is a common practice among practitioners of cryo-EM. This does not mean that formyl substituents can never be resolved in ESP map—they certainly can be at adequate resolutions. This is nicely exemplified in the recent 2.4 Å global resolution structure of FRL-PSI from Kato et al.^3^ in which the formyl substituents of some Chl *f* molecules can be directly observed in the highest-resolution regions of the map, ~2.3 Å. However, other assignments are far less convincing, as described below. This implies that the resolution required to observe the formyl substituents of Chls *b*, *f*, and *d* directly in cryo-EM-derived ESP maps is ~2.3 Å, a value that has only rarely been achieved in published structures. Currently, however, a formal resolution “cutoff value” to assign negatively or partial negatively charged atoms unambiguously does not exist.

## Modeling discrepancies in Chl *b*- and *f*-containing structures

### Chl *b*-containing sites from LHCI

Resolution limitations are important to consider when assigning different Chl molecules regardless of technique. In plants and algae, LHCs are associated with PSI and PSII. Two of the major LHCs are LHCI and LHCII, both of which contain Chl *a* and Chl *b* molecules. Two crystal structures of PSI with four associated LHCI peripheral antenna complexes were reported for *Pisum sativum* (pea) at 2.8 Å resolution in 2015^40,41^ and a refinenment of one to 2.6 Å resolution was subsequently reported in 2017^42^. In the initial two structures, nine Chl *b* sites were assigned in one (PDB 4Y28) and the same nine Chl *b* sites plus three additional Chl *b* sites were assigned in the other (PDB 4XK8). The former structure erred on the side of caution, assigning Chl *b* only when the formyl substituent was unequivocally identified in the electron density map. The drawback of this approach, however, is that it probably underestimates the true Chl *b* content. X-ray data at 2.8 Å resolution are near the limit for being able to identify the formyl substituent directly, especially in more flexible regions with higher B-factors. Indeed, nine Chl *b* molecules per complex was less than the number expected from prior pigment analysis estimates^43^. The Chl *b* assignments of Qin et al.^41^ agreed with their pigment analysis based upon solvent extraction and also agreed with the analysis performed previously^43^, so it would have been reasonable to search for a specific number of Chl *b* molecules (12 in this case). The drawback of this approach, however, is that some of the Chl *b* assignments are less confident due to resolution limitations. Both approaches are reasonable for making Chl *b* assignments based on the density map, and because nine Chl *b* assignments were common to both structures, these logically were assumed to be the most confident assignments. Mazor et al.^42^ subsequently reported in 2017 the structure of pea PSI-4LHCI at slightly higher resolution, 2.6 Å (PDB 5L8R). The increased resolution allowed the authors to observe the formyl substituents on Chl *b* directly and 13 Chl *b* molecules were assigned to specific sites. These sites included all nine of the most confidently assigned Chl *b* binding sites, the three additional sites from Qin et al.^41^, and one additional site. Thus, ~2.6 Å resolution X-ray diffraction data were required for direct observation of all 13 Chl *b* formyl groups in the PSI-4LHCI complex (assuming that 13 is indeed the actual number of Chl *b* bound in these complexes). Notably, 2.6 Å is also the commonly accepted lower-resolution limit for confident assignment of water molecules by X-ray crystallography.

Cryo-EM structures should have an increased resolution requirement for direct observation of the formyl group on Chl *b* due to the partial negative charge on oxygen, which causes the oxygen to contribute less to the ESP map. There are currently five cryo-EM structures of PSI complexes containing LHCI: one from maize^8^ and four from *Chlamydomonas reinhardtii*^4,6^. The structure of maize PSI with one LHCII trimer and four LHCI complexes was solved to 3.3 Å resolution (PDB 5ZJI)^8^. As the maize structure has a resolution of only 3.3 Å, the authors apparently assigned 12 Chl *b* positions in LHCI solely on the basis of the 12 common Chl *b* positions in the 2 X-ray structures from *P. sativum*^40,41^, even though one of those structures actually contains 13 assigned Chl *b* molecules and neither is derived from maize, which could produce LHCI complexes that bind a different number of Chl *b* molecules.

Four structures of PSI with multiple LHCI complexes were presented in 2019 at similar resolutions but using two very different approaches for assigning Chls. The structures of *C. reinhardtii* PSI with eight and ten LHCI complexes were reported at 2.89 Å and 3.3 Å resolution^6^, respectively (PDB 6IJJ and 6IJO, respectively). Chl *b* molecules were not assigned because of the limited resolution for both structures, especially because most or all of the Chl *b* molecules are expected to be located on the most peripheral LHCI belt, where the local resolution is likely to be lower than the reported global resolution. No PSI-LHCI complex in this arrangement has previously been solved by X-ray crystallography nor from this organism. Thus, it was not possible to assign the Chl *b* molecules in these complexes and therefore the authors modeled all Chls as Chl *a*. This importantly and clearly shows that, without prior knowledge, none of these Chls could confidently be assigned at these resolutions.

Another study reported the same two structures, PSI-8LHCI and PSI-10LHCI, from *C*. *reinhardtii* at 2.8 Å and 3.3 Å resolution^4^, respectively (PDB 6JO6 and 6JO5; the former is reported in the Protein Data Bank as 2.9 Å resolution). Seventeen Chl *b* molecules were assigned in the PSI-8LHCI structure and 19 Chl *b* molecules were assigned in the PSI-10LHCI structure. The authors mention it being “impossible in principle” to differentiate Chl *a* and *b* at the resolution of their data; however, they made assignments based on potential H-bonding interactions with the formyl substituent of Chl *b*. This major difference in approach to assigning Chl *b* highlights the potential pitfalls of requiring higher resolution to make such assignments confidentally in cryo-EM ESP maps. As these examples illustrate, authors must make clear what criteria are being employed to make functionally important assignments of Chls in their structures.

### Chl *b*-containing sites from LHCII

LHCII complexes are peripheral antenna proteins containing Chls *a* and *b*, and carotenoids, which are primarily associated with PSII in plants and green algae. An electron crystallographic structure of *P. sativum* LHCII was determined in 1994 at 3.4 Å resolution, which was insufficient to differentiate the two Chl types, although tentative assignments were made on the basis of spectroscopic evidence^44^. Ten years later, direct observation of C7 formyl substituents was possible in the 2.7 Å resolution X-ray crystal structure of spinach LHCII, in which 6 of the 14 total Chl sites were assigned as Chl *b*^45^. Subsequently, five of these six Chl *b* sites were confirmed when the 2.5 Å resolution crystal structure (PDB 2BHW) of *P. sativum* LHCII was reported in 2005^46^; the authors speculated that the sixth might be a mixed Chl *a*/*b* site because of missing density for the C7 formyl substituent and the expected Chl *a*/*b* ratio from pigment analysis. Notably, this sixth Chl *b* was the only one without an obvious H-bonding interaction to its formyl sidechain, although this substituent is situated near the solvent-exposed periphery of the complex and solvent water could fulfill this role.

Various LHCII-containing cryo-EM structures have been published since then^6,8,10–12,24,25^. The first such structure of a spinach PSII-LHCII supercomplex at 3.2 Å resolution appeared in 2016 (PDB 3JCU)^12^. Six Chl *b* sites were assigned per LHCII solely on the basis of the 2.7 Å resolution X-ray crystal structure of LHCII from spinach, because the resolution was insufficient to observe the formyl substituents of Chl *b* directly. Indeed, all of the cryo-EM structures containing LHCII have based their Chl *b* (or even Chl *c* in structures from a diatom where the C8 position may exhibit different substituents^47^) assignments based solely on prior spinach LHCII X-ray crystal structures rather than by direct observation. The fact that all of the cryo-EM structures of LHCII but one^12^ are not from spinach but are instead from *Arabidopsis thaliana*^11^, *P. sativum*^10^, *Zea mays*^8^, the green alga *C. reinhardtii*^21^, and a diatom, *Chaetoceros gracilis*^24,25^ means the assignments do not account for potential species-specific variation. The assignment problem is further exacerbated, because many of the LHCII complexes are found in peripheral locations of the supercomplexes. The local resolution is usually substantially lower for these peripheral complexes than the global resolution and this puts the target resolution required for direct observation even further out of reach. Although such assignments are reasonable as a starting point, they can only be considered tentative and unconfirmed. Failure to recognize this point, or to clearly enunciate it to readers of papers, could cause some to misinterpret the results during attempts to use the results for energy-transfer calculations and other downstream applications of the structures.

### Chl *f*-containing sites from FRL-PSI

FRL photoacclimation, or “FaRLiP”^48^, confers the ability for cyanobacteria to absorb FRL^49^, the energy of which is converted to chemical energy by their photochemical oxidoreductases PSI and PSII. FaRLiP allows organisms to thrive in shaded environments where FRL is prevalent, such as soils^50^, microbial mats^51^, photic zones of caves^52^, stromatolites^53^, and biofilms associated with beachrock^54,55^. Cyanobacteria are especially important primary producers of organic carbon^56^; therefore, understanding the way in which they have adapted and can acclimate to various environments provides insight into novel biological mechanisms that may be used to engineer FRL absorption into oxygenic phototrophs that generally grow slowly in shaded environments^57,58^.

On the molecular scale, changes are observed in light-harvesting protein complexes including the phycobilisome, PSI, and PSII^48,49,59–63^. It is well-established that PSI in commonly studied model cyanobacteria contains only Chl *a* molecules^23,64,65^; however, as a result of FaRLiP, some cyanobacterial species express six alternate subunits that accommodate the incorporation of ~8% Chl *f* into PSI^2,3,59,66–69^. Understanding the energetic landscape of the photosystems is difficult when each pigment is the same Chl type and individual sites are almost indistinguishable spectroscopically. Therefore, the unambiguous assignment of Chl *f* sites in FRL-PSI would be advantageous, because it may help to deconvolute the PSI energy-transfer landscape.

Cryo-EM structures of FRL-PSI from *Fischerella thermalis* PCC 7521^2^ and *Halomicronema hongdechloris*^3^ were recently published. Each assigns the positions of important Chl *f* molecules, but unlike the case for the recent LHC structures, there are no previous structural data on which to base Chl assignments. As the labeling of the Chl molecules modeled in the two structures is different, Supplementary Table 1 gives the antenna Chl site nomenclature conversion for the assignments described in the two FRL-PSI structural studies. The labels used by Gisriel et al.^2^ maintain the convention first introduced by Jordan et al.^64^, which allows for consistent site naming among different PSI structures.

**Fig. 2:**
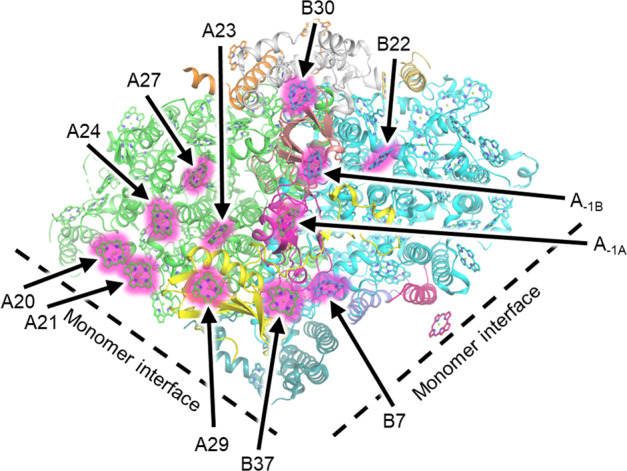
Proposed Chl *f* binding sites in FRL-PSI. The structure of PSI from *Thermosynechococcus elongatus*^64^ that initially devised a useful nomenclature for Chl sites in PSI is shown. Although seven Chls *f* are expected per FRL-PSI monomer, 12 sites have been proposed from various investigations (pink glow). Only a single monomer of the PSI trimer, secondary structures of protein subunits, and tetrapyrrole rings are shown for clarity.

Various investigations have used spectroscopic approaches to identify the specific Chl *f* sites within FRL-PSI^29,30,67,70–72^; however, these have resulted in some conflicting assignments that will be discussed below. Figure 2 shows a monomer of PSI and each of the 12 positions that have been proposed to be Chl *f* in the current literature—although each monomeric complex should only contain ~7 Chl *f* molecules (8% of ~90 total Chls). Spectroscopic studies have provided evidence for^29,70–72^ and against^30^ the presence of Chl *f* in one or both A_–1_ sites in the electron-transfer chain (ETC), although neither of the two recent cryo-EM structures assigned any Chl *f* molecules in the ETC^2,3^. Gisriel et al.^2^ assigned four Chl *f* sites in the core antenna of the FRL-PSI structure from *F. thermalis* PCC 7521 and Kato et al.^3^ assigned seven Chl *f* sites in FRL-PSI from *H. hongdechloris*, but only one position was commonly identified in the two structures. This discrepancy clearly highlights the challenge of distinguishing very minor yet functionally critical structural differences in the context of a protein complex with a mass of more than 1.1 MDa and more than 100 total cofactors. Although this is already an extreme case, some photosynthetic complexes studied to date by cryo-EM have masses of ~15 MDa, more than 700 protein subunits, and nearly 1600 isomeric bilin chromophores belonging to three chemical types^17,18^. Distinguishing small chemical differences on highly similar cofactors can have tremendous functional significance.

The two cryo-EM structures of FRL-PSI are derived from different cyanobacterial species, as noted above, and are reported at global resolutions of 3.19 Å (PDB 6PNJ, Gisriel et al.^2^) and 2.4 Å (PDB 6KMX, Kato et al.^3^), respectively. The local resolution of Chl *f*, specifically its defining formyl substituent at the C2 position in the tetrapyrrole ring (Supplementary Fig. 1), ultimately determines the ability to differentiate it directly from Chl *a*, which has a methyl substituent at the C2 position. Gisriel et al.^2^ note that the resolution of their cryo-EM density map is not sufficient to allow direct observation of this difference and they based their assignments on nearby potential H-bonding donors to formyl substituents that are unique within the FRL-PSI structure. Kato et al.^3^ determined structures of both white-light (WL)-PSI and FRL-PSI from *H. hongdechloris* and assigned Chl *f* in FRL-PSI based on: (a) the identification of unaccounted-for density at the C2 position using an algorithm from the Phenix software suite^73^ when each tetrapyrrole was modeled as Chl *a*; and (b) by subtracting the density of each Chl from the WL-PSI map from the corresponding density of the FRL-PSI map. In both cryo-EM structures, some assignments are more convincing than others. However, the fact that only one of the initially assigned Chl *f* sites agrees between the two structures demonstrates the inadequacy of current methods and misunderstandings concerning the challenges for making such assignments using cryo-EM-derived density maps.

## Local chemical environment of Chl formyl substituents

### Lessons from examining Chl *b* in LHCI and LHCII

**Fig. 3:**
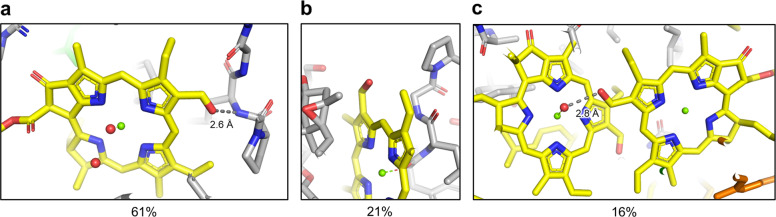
Examples of common Chl *b* sites from the LHCII X-ray crystal structure. All panels show the 2.5 Å X-ray crystal structure of *P. sativum* LHCII (PDB 2BHW)^46^. **a** H-bonding from the C7 formyl substituent may be directly from an H-bonding donor such as a backbone amide nitrogen. **b** H-bonding to the formyl substituents of Chls *b* may not be observed. However, note the large volume available for solvent water access to the formyl oxygen. **c** When a *π*-stacked dimer of Chl *b* occurs, one formyl substituent receives an H-bond from a water molecule that is also providing the axial ligand of the other Chl *b* in the dimer.

An important consideration when attempting to identify the formyl substituents of Chls is their chemical environment. As X-ray crystal structures of LHCI and LHCII are available, these data present an opportunity to examine the chemical environments of the formyl groups of Chl *b* molecules to establish guidelines for their accurate assignment in ESP maps near or below the resolution required for direct observation. The two X-ray crystal structures include nineteen total Chl *b* sites: thirteen from LHCI and six from LHCII. Of these 19 sites, 7 lack obvious H-bonding to the C7 formyl substituent of the Chl occupant. Of the seven Chls that lack H-bonding to the formyl substituent, three (two in LHCI and one in LHCII) are found in *π*-stacked Chl *b* dimers, in which the Chl *b* lacking the H-bond to its formyl substituent is axially coordinated by a water molecule; the formyl group of the other Chl *b* molecule in the dimer accepts an H-bond from that axially coordinating water. These observations suggest three general motifs that coordinate Chl *b*, which are listed here from most to least common: (1) the C7 formyl substituent of Chl *b* serves as an H acceptor from a water molecule or a protonated amino acid (this is observed for 12 of the 19, or 61%, of Chl *b* sites in the 2 structures); (2) the C7 formyl substituent of Chl *b* may exhibit no obvious H-bonding partner (observed for 4 of the 19, or 21%, of Chl *b* sites in the 2 structures; (3) a *π*-stacked dimer of Chl *b* molecules may exhibit 1 stable H-bond to 1 formyl substituent but may not exhibit an H-bond to the second (observed in 3 of the 19, or 16%, of the Chl *b* sites in the 2 structures). Figure 3 shows an example of each of these motifs from the LHCII X-ray crystal structure. It is important to consider, however, that the frequency of the second category (no obvious H-bond exists to the formyl substituent) may be misleading, because unmodeled water molecules could exist in the structure. This might be especially common in regions with slightly higher B-factors near the periphery of the structure, which is the case for all four of the sites of this category. It is further worth noting that, because of the extreme structural similarity between Chl *a* and Chl *b*, there would be no site selectivity if no stabilizing interaction (e.g., H-bond) occurred to differentiate Chl *a* from Chl *b* at the molecular level. Logically, if a site has an occupancy of Chl *b* sufficiently high to be assigned as such in the X-ray crystal structure, there must be an underlying chemical basis for the selective binding of Chl *b* over Chl *a* at that site.

### Chl *f* binding sites in FRL-PSI may be informed by the Chl *b*-site analysis from LHCs

The observations regarding Chl *b* coordination in LHC complexes are useful when assigning Chl *f* molecules in the FRL-PSI experimental ESP maps in which the local resolution may be insufficient to make their assignments confidently. Model features should be more reliable in regions closer to the center of the trimeric PSI structures where local resolution is highest. Water molecules are modeled in the structures from Kato et al.^3^, because those maps have sufficient resolution to locate them in certain regions. We wish to emphasize that Chl *f* molecules are probably less likely to occur naturally in nonspecific binding sites than Chl *b* molecules because energy transfer from Chl *b* to Chl *a* is energetically downhill, but energy transfer from Chl *f* to Chl *a* is energetically uphill. Therefore, Chl *f* probably exhibits greater site specificity to avoid low energy traps at physiological temperature that could greatly lower the overall quantum efficiency of using FRL to power oxygenic photosynthesis. Nature has evolved to employ a complex set of FRL-specific, paralogous proteins precisely to avoid this possibility.

**Fig. 4:**
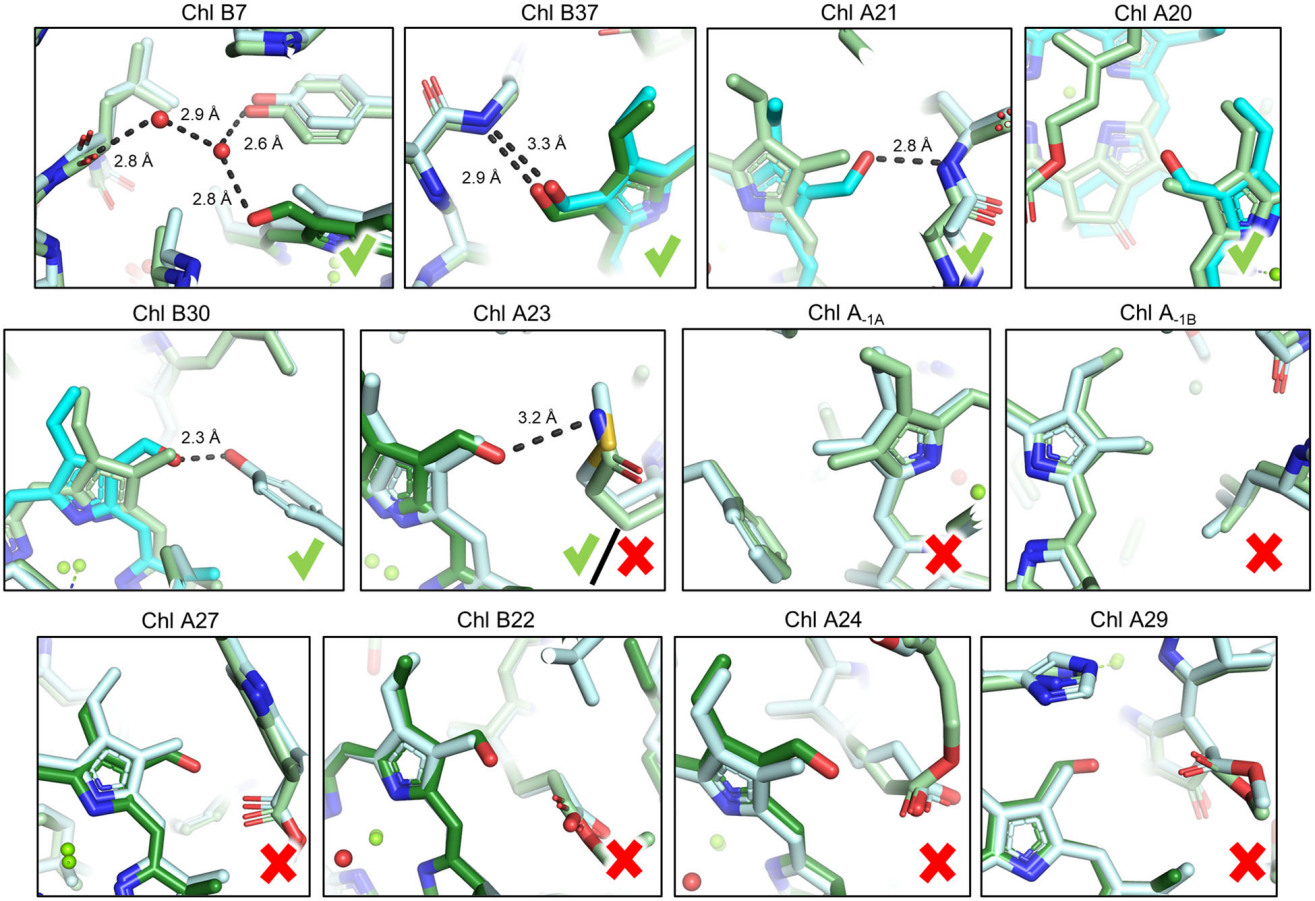
Chemical environment of 12 proposed Chl *f* sites. The name of each site (Supplementary Table S1) is listed above the image. Possible H-bonding interactions are shown as black dashes. Green checks designate those environments that agree with observations in the LHCII Chl *b* chemical environment analysis (Fig. 3) and red X’s do not. The Chl A23 panel with both the check and the X pertains to a site that could be species-dependent, as discussed in the main text. The structure from *F. thermalis* is shown in light blue with Chl *f* colored cyan and the structure from *H. hongdechloris* is shown in light green with Chl *f* colored dark green.

**Fig. 5:**
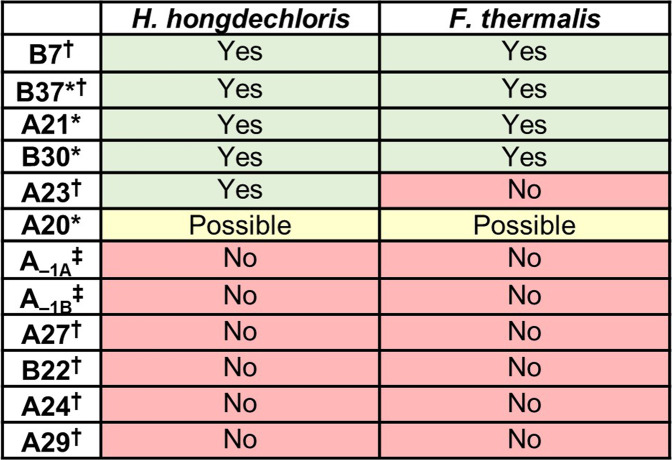
Presence or absence of evidence for H-bonding to C2 substituents of the 12 previously proposed Chl *f* sites in FRL-PSI complexes. The positions shown in Fig. 4 are listed and the presence of evidence (yes/no) for being Chl *f* based on the LHC analysis is indicated for each FRL-PSI structure. An asterisk (*) denotes that the Chl was originally assigned as Chl *f* in the structural model of Gisriel et al.^2^; a dagger (†) denotes positions that were originally assigned as Chl [f] in the structural model of Kato et al.^3^; and a double dagger (‡) denotes positions that were proposed to be Chl *f* on the basis of spectroscopic data from Nürnberg et al.^29^.

The local environments of all currently proposed Chl *f* molecules (Fig. 2) in FRL-PSI are shown in Fig. 4 and a thorough discussion regarding each prospective position can be found in Supplementary Text 2. Chls B7, B37, A21, and B30 can be confidently assigned as Chl *f* in both FRL-PSI ESP maps from Kato et al.^3^ and Gisriel et al.^2^, because their formyl position is consistent with the first observation from the LHC analysis above that each clearly accepts an H-bond from the polypeptide or a water molecule. The A21 and B37 Chl *f* sites are both members of a dimer together with a Chl assigned tentatively as Chl *a* in both FRL-PSI structures, which are Chl A20 and B38, respectively. Spectroscopic results suggest the presence of a Chl *f* dimer^29,30^, making Chls A20 and B38 strong candidates to also be Chl *f*, although neither obviously exhibits H-bonding to their formyl substituents in the two structures. However, the resolution of the ESP map may limit such an assignment, e.g., by masking the presence of H-bonded water molecules. If these Chls are not H-bonded, it does not rule out their possible Chl *f* identity, which would follow the third observation regarding the Chl *b* analysis in LHC that a *π*-stacked dimer of Chl *b* molecules may exhibit one stable H-bond to one formyl substituent but may not exhibit an H-bond to the other. Finally, the A23 site in *H. hongdechloris* is probably Chl *f*, but it is not in *F. thermalis* PCC 7521, exemplifying the possibility of species-specific Chl *f* sites. Importantly, our analysis suggests that there is no compelling evidence that Chls A_–1A_, A_–1B_, A27, B22, A24, or A29 are Chl *f*. These observations are summarized in Fig. 5.

## Outlook for Cryo-EM in photosynthesis research

**Fig. 6:**
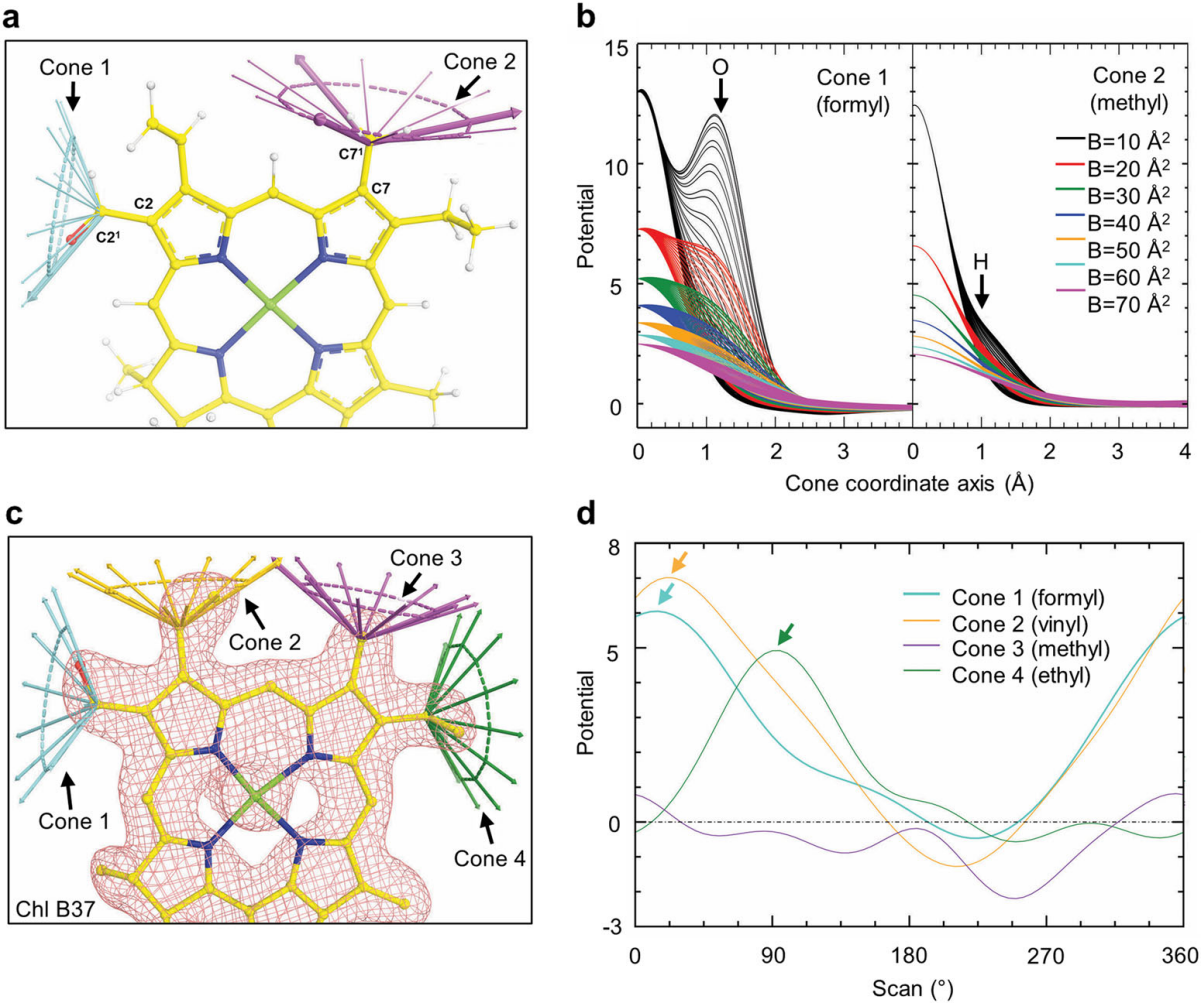
Quantitative analysis of density features for substituents on Chl tetrapyrrole rings. **a** The C2 and C7 substituents of a calculated Chl map can be analyzed by recording the density along a series of axes at an angle expected for the substituent’s bonds (formyl oxygen = 120° from the C2-C2^1^ axis in blue, protons = 109.5° from the C7-C7^1^ axis in purple). **b** Plot of ESP density either 1.2 Å or 1.0 Å away from the C2/7^1^ atom at 120° and 109.5°, respectively. This plot is shown for various B-factors that correspond to possible local resolutions. Note that the density contribution of the atoms bonded to CX^1^ disappears at higher B-factors. **c** Chl B37 and its corresponding density from the Kato et al.^3^ FRL-PSI ESP map at 5*σ* where the color of the cones corresponds to **d**. **d** A scan of four substituents from **c** using formyl parameters (120° at 1.2 Å) about the CX-CX^1^ bond axis. It is noteworthy that the methyl substituent exhibits no peak, the ethyl substituent exhibits a peak ~90° (green arrow, not in plane with the tetrapyrrole ring), and both the formyl and vinyl substituents exhibit a peak ~0/360° (cyan and yellow arrows, respectively, and in plane with the tetrapyrrole ring). The density for the partial negatively charged oxygen of the formyl substituent is lower than that of the neutrally charged carbon atom of the vinyl substituent as expected.

Cryo-EM is a powerful technique that often allows molecular structures of macromolecules to be solved with much less effort than X-ray crystallography. However, the resolution achieved by cryo-EM is currently lower than that typically achieved by X-ray crystallography. Although some low-resolution cryo-EM structures can be informed by previously solved higher-resolution structures, it is important that authors communicate clearly that such assignments are based on analogy and not upon direct observation. When no previous structure is known, modeling of functionally important cofactors should be done cautiously and by taking into account the chemical environment of the ligand. This is exemplified by the fact that only 4 or 5 of Chl *f* sites of the 12 that have been proposed in the literature^2,3,29^ exhibit sufficiently convincing evidence to actually be assigned as Chl *f* in each FRL-PSI cryo-EM structure (Fig. 5) and 6 of those that have been assigned are probably Chl *a* instead. The observations discussed here should serve as a warning to all structural biologists using cryo-EM: small substituents in cryo-EM maps, especially those exhibiting negative or partial negative charges, may be extremely difficult to resolve; careful consideration of the chemical environment and awareness of potential artifacts in difference maps that arise from local B-factor differences are imperative in making correct assignments. We note that even if Chl A20 or B38 is Chl *f* in these structures (and if Chl A23 is Chl *f* in *F. thermalis* FRL-PSI), this would still leave one or two Chl *f* molecules unassigned according to bulk pigment estimates^2,3,29,59,66–69^. It is possible but unlikely that the equivalent of one or two extra Chl *f* molecules observed by bulk cofactor analysis may not bind to specific sites, but may rather bind promiscuously to many Chl sites where locally there is space for the formyl substituent to fit, probably near the peripherary, as is exhibited by the Chl *b* in Fig. 3b. Such binding of Chl *f* to nonspecific sites has already been demonstrated to occur by Kurashov et al.^67^ and the bound Chl *f* molecules in that case were partly functional in light harvesting^74^. However, they did not exhibit such extreme far-red absorbance and high relative quantum yield for promoting photochemistry as observed in natural FRL-PSI complexes.

The multitude of cryo-EM structures emerging in the literature recently has profoundly contributed to our understanding of the diversity of phototrophy on the molecular scale and this trend appears likely to continue and even expand. There are multiple Chl types in other reaction centers, photosystems, and antenna complexes, such as those found in FRL-PSII^29,75^, the photosystems of *Acaryochloris marina*^58,76^, *Candidatus Chloracidobacterium (Cab.) thermophilum*^77^, and possibly others not yet discovered. Robust methods to distinguish Chls in cryo-EM density maps will be required to identify their positions. Presently, we find 16 Chl, bacteriochlorophyll, or pheophytin variants that have been found as ligands in the PDB, but this is only a fraction of those variants that are known to exist^27,78–80^ and do not have an associated molecular structure. As cryo-EM continues to provide molecular structures of photosynthetic membrane protein complexes, more variants will be deposited into the PDB. The differences between most of these variants are small and therefore photosynthetic research could benefit from developing new quantitative methods for robust identification of small substituents on tetrapyrrole rings in cryo-EM maps at medium resolution. These structural issues extend beyond Chls to carotenoids, which exhibit far greater structural diversity and for which the presence of keto or hydroxyl groups can alter their chemical properties quite significantly^81^. It is further worth noting that the issues discussed here almost certainly extend beyond proteins directly involved in photosynthetic light harvesting and electron transfer. Chls and carotenoids have been shown to be present in other enzymes (e.g., the cytochrome *b*_6_*f* complex^82,83^, lycopene cyclase^84^, and ferrochelatase^85^). Finally, issues similar to those raised here could be imagined in the case of heme *o* and heme *a*, which also only differ by a methyl/formyl group substitution, and which sometimes seem to exchange in organisms depending on growth conditions^86^.

Polypeptides are relatively easy to model at the resolutions currently being routinely achieved by cryo-EM, because the sequence is typically known and one can confidently assign larger residues such as phenylalanine that essentially anchor smaller residues nearby in the chain that are more difficult to assign. For tetrapyrroles, however, this advantage is not possible as is evident by our analysis of Chls *b* and *f* herein. With these considerations in mind, we suggest an approach for quantitatively assessing cryo-EM density maps to fit small substituents of tetrapyrroles. It is assumed that the coordinates of the tetrapyrrole ring atoms of Chls can be placed with reasonable accuracy and thus the densities associated with substituents can be quantitatively assessed (Fig. 6). If density for a formyl substituent is examined along a circle traced about the CX-CX^1^ bond axis, where X corresponds to the position on the tetrapyrrole ring, at 120°, we would expect a single peak ~1.2 Å from the CX^1^ atom, the expected CX^1^ = O bond length, corresponding to the density for an O atom (Fig. 6a,b). If the same analysis is performed using a methyl substituent at very high resolution (low B-factor), three weak peaks should appear at an angle of 109.5° from the axis ~1 Å, from the CX^1^ atom, corresponding to three H atoms (Fig. 6a,b). However, the local resolution will determine the amplitude of the peak(s), where large B-factors show peaks much more difficult to distinguish (or not at all) than for smaller B-factors. This highlights the importance of reporting local resolution where these formyl substituents are modeled or at least making half maps publicly available.

When searching specifically for a formyl substituent among the tetrapyrrole substituents in an experimental ESP map using this method, a scan around a cone angled at 120° and 1.2 Å from the CX-CX^1^ bond axis can be performed as described above, and if the map exhibits sufficiently high local resolution, the above observation shows that a peak should be found nearly co-planar to the tetrapyrrole ring. As a proof-of-principle, we show this analysis in the ESP map of Kato et al.^3^ for the most convincing Chl *f* assignment, Chl B37, which is assigned as Chl *f* in both FRL-PSI structures, in Fig. 6c,d. At least in this region of the map where the local resolution is likely the highest, around 2.3 Å, this expectation is confirmed. Whereas the methyl substituent does not appear to exhibit a substantial peak, the ethyl substituent exhibits a peak out of plane with the tetrapyrrole ring, and the vinyl and formyl substituents exhibit peaks in plane with the tetrapyrrole ring. Due to the partial negative charge on the oxygen atom of the formyl substituent, however, its peak is slightly smaller than that of the vinyl substituent. Therefore, we conclude that both the ESP map density and the local chemical environment (described above) support the assignment of B37 as Chl *f*.

We can further quantify the reliability and the confidence level of such an assignment by statistically establishing a noise threshold upon analyzing the signals corresponding to a formyl substituent in a number of Chls in a given EM map. Although the density profile for the methyl substituent in the same ring may partly serve as an internal control for differentiating the signal of the oxygen group of the formyl moiety from noise at a similar local resolution, a more thorough analysis of many tetrapyrroles at various resolution shells would be desirable. In any case, an analysis of this type, in conjunction with an evaluation of the chemical environment as we have described above, could significantly improve cofactor assignments when attempting to identify such difficult-to-resolve substituents in the future.

## Supplementary information

Supplementary Information
