# Supplementary material for: Opportunities and challenges for assigning cofactors in cryo-EM density maps of chlorophyll-containing proteins
Source: Commun Biol. 2020 Jul 30;3:408. doi: 10.1038/s42003-020-01139-1 (PMC7393486; doi:10.1038/s42003-020-01139-1)
Supplement: Supplementary file 1 — Supplementary Information [file 42003_2020_1139_MOESM1_ESM.pdf]

Supplementary Information for

Opportunities and challenges for assigning cofactors in cryo-EM density maps of chlorophyll-containing proteins

Christopher J. Gisriel[1,*], Jimin Wang[2], Gary W. Brudvig[1,2], and Donald A. Bryant[3,4]
[1]Department of Chemistry, Yale University, New Haven, CT 06520, USA.
[2]Department of Molecular Biophysics and Biochemistry, Yale University, New Haven, CT 06520, USA.
[3]Department of Biochemistry and Molecular Biology, The Pennsylvania State University, University Park, PA 16802, USA.
[4]Department of Chemistry and Biochemistry, Montana State University, Bozeman, MT 59717, USA.
*To whom correspondence should be addressed: christopher.gisriel@yale.edu

Supplementary Text 1.

Supplementary Text 2.

Supplementary Figure 1. The four major Chls of cyanobacteria and higher plants.

Supplementary Figure 2. Sequence alignments supporting Chl B30 as Chl *f* in both *H. hongdechloris* and *F. thermalis* FRL-PSI.

Supplementary Figure 3. Sequence alignments supporting Chl A23 as a species-dependent Chl *f* site.

Supplementary Figure 4. Environment of Chl A_{-1} sites.

Supplementary Table 1. Nomenclature conversion table for the FRL-PSI structure manuscripts.

Supplementary Text 1

Basis for electric potential. Electric potential maps measured by cryo-EM can be thought of as the product of the convolution of the electric potentials of the atoms in molecules when they are in their average positions with their relative motions. The electric potential $\psi(r)$ is the energy required to move a positive test charge from infinity to the location in space designated by the vector r. The electric potential of a proton for example is given by the following equation.

$$\psi(r) = q/4\pi r\varepsilon, \qquad \text{(Equation 1)}$$

where q is the charge in Coulombs, ε is the permittivity of the medium in which the charge is situated, r is distance in meters, and the potential is in volts.

For any atom, the nuclei generate a positive potential, and the electrons generate a negative potential, both of which are measured together as a combined property of the atoms of molecules in a cryo-EM structure. For any neutral atom, the distributed electrons of the atom completely shield the positive potential of the nuclei within its van der Waals radius, which is commonly called the van der Waals potential. For any ionized atom, the electric potential can be thought of as the sum of the van der Waals potential and the Coulomb potential associated with the net atomic partial charges of the atom. For comparison, X-ray crystallography measures only distributed electrons, and neutron crystallography measures only the nuclei.

Supplementary Text 2

Evidence that Chls B7, B37, A21, and B30 are Chl *f* in both FRL-PSI complexes. Each of these Chl sites obeys the first and most convincing observation from the LHC analysis (**Fig. 3a**). In each case, the C2 formyl substituent is appropriately positioned to accept an H-bond from an amino acid sidechain, peptide amide nitrogen, or water molecule. The inability to resolve water molecules in the structure of Gisriel *et al.*[1] caused the misassignment of Chl B7 as Chl *a*; however, the conserved position of the protein environment, especially the hydroxyl group of the nearby Tyr sidechain, argue that this site is indeed Chl *f* in the FRL-PSI complexes from both organisms. Chl B37 was assigned as Chl *f* in both structures due to readily apparent, direct H-bonding between an amide N of the protein backbone with the of the substituent at position C2 of the tetrapyrrole. Chl A21 was modeled as Chl *f* in the structure from Gisriel *et al.*[1] but not in Kato *et al.*[2]; however, the formyl O exhibits an ideal H-bonding distance to a backbone amide of a loop in the PsaA2 polypeptide, making it highly likely to be Chl *f*.

The FRL-PSI structure from Kato *et al.*[2] is missing two peripheral subunits that are FRL-specific: PsaJ2 and PsaF2[3]. Although the authors observed these subunits in sodium dodecyl sulfate-polyacrylamide gel electrophoresis analysis, their occupancy was too low to be modeled in the 3D reconstruction, leading the investigators to suspect that these subunits are loosely bound and partially dissociate during cryo-EM sample preparation. In the FRL-PSI structure from *F. thermalis*, both subunits are present, and Chl B30 is assigned as Chl *f* because its C2 substituent is positioned to accept an H-bond from the hydroxyl moiety of a nearby Tyr sidechain, which is specific to PsaJ2, the FRL-isoform of the PsaJ subunit, in *F. thermalis* and other FaRLiP strains. The corresponding Chl is also likely to be Chl *f* in *H. hongdechloris* because the Tyr present near the C-terminus is conserved in the PsaJ2 sequence of that organism, which differs from PsaJ1 as shown in **Supplementary Fig. 2**. Thus, the replacement of PsaJ1 by PsaJ2 during FaRLiP introduces a distinct functional difference, the ability to coordinate Chl *f*, by replacing the PsaJ1 subunit expressed in WL-PSI. As pointed out above, this is precisely why nature has evolved the use of paralogous proteins to utilize FRL efficiently.

Chl A20 may be Chl *f* in a π-stacked dimer with Chl A21. Chl A21, which is confidently assigned here as Chl *f*, is in a π-stacked dimer with Chl A20. In the structure from Kato *et al.*[2], the isoprenoid tail of Chl A21 wraps onto the surface of its tetrapyrrole ring, which might reside near the C2 substituent of Chl A20. Because this positioning of the isoprenoid tail would seemingly disfavor a formyl group at C2, this suggests that Chl A20 should be Chl *a*. On the other hand, while this tail orientation is not unusual among Chls, this region probably exhibits lower resolution because it is both flexible and solvent-exposed, thereby decreasing the confidence of the tail position in the model. Note that Chl *b* dimers make up ~16% of the Chl *b* molecules in two LHC X-ray crystal structures. If Chl A20 is Chl *f*, the interactions of the formyl group are ambiguous, because it is located near a solvent-exposed space at the monomer-monomer interface. Thus, although the evidence is weaker than for Chls B7, B30, B37, and A21, Chl A20 is possibly Chl *f*. Spectroscopic evidence[4,5] strongly suggests that a Chl *f* dimer exists in FRL-PSI from both *Chroococcidiopsis thermalis* PCC 7203 and *F. thermalis* PCC 7521 FRL-PSI, and the same is probably true for *H. hongdechloris*. At 77 K, FRL-PSI from *F. thermalis* exhibits a weak emission band at 806 nm that is likely to be associated with a Chl *f*/Chl *f* dimer, while most of the emission is observed at 746 nm, which likely arises from monomeric Chl *f* or Chl *f*/Chl *a* heterodimers. The spectroscopic results could also possibly be interpreted as arising from a Chl *a*/Chl *f* heterodimer. In either case, the Chl A20/A21 and ChlB37/B38 dimers are the most likely candidates to be this

spectroscopically distinct dimer because each contains at least one Chl *f*. Further spectroscopic studies, combined with site-specific mutagenesis, should resolve these issues.

Chl A23 may represent a species-specific Chl *f* site in *H. hongdechloris*. Supplementary Fig. 3 shows sequence alignments comparing polypeptides from both *F. thermalis* and *H. hongdechloris* that are implicated in H-bonding to the C2 substituent of Chl A23. In the FRL-PSI structure from Kato *et al*.[2], Chl A23 is confidently assigned as Chl *f*, because it exhibits a direct H-bond to the amide group of a nearby Gln residue. They further show that this Gln residue is conserved in two other FRL-PSI species, suggesting that it might be a conserved feature and FRL-specific. However, a methionine replaces this glutamine in all *Fischerella* species, implying that species-specific variation may occur for Chl *f*-binding sites. Whether this implies that there are differences in the total number of Chl *f* molecules bound to FRL-PSI complexes of different FRL-PSI complexes remains to be determined.

Evidence against Chls A_{-1A}, A_{-1B}, A27, B22, A24, A29 being Chl *f*. While spectroscopic analyses have suggested that one or both A_{-1} sites are Chl *f* in *C. thermalis*[4,6,7] and *F. thermalis*[8], neither of the structures of FRL-PSI in *F. thermalis* or *H. hongdechloris* assign these Chls as Chl *f*. It is possible that *C. thermalis* has Chl *f* in one or both A_{-1} sites, which would represent a species-specific difference from *F. thermalis* or *H. hongdechloris*. However, the sequences of PsaA2 and PsaB2 among these three organisms in the corresponding regions are very strongly conserved, which contradicts this hypothesis. A superposition of the two FRL-PSI cryo-EM structures[1,2] is shown in **Supplementary Fig. 4** together with a homology model of FRL-PSI from *C. thermalis*, all of which suggest that all residues nearby the C2 substituent of the A_{-1} sites are well conserved and that there are no nearby H-bond donors. Furthermore, Cherepanov *et al*.[5] have studied FRL-PSI from *F. thermalis* PCC 7521 by ultrafast pump-probe laser spectroscopy and concluded that there are no Chl *f* molecules in any positions among the electron-transport chain cofactors of these FRL-PSI complexes. The present evidence strongly indicates that all Chl *f* molecules occur in the antenna regions of FRL-PSI and that Chl *f* does not play a role in electron-transfer processes in these complexes.

Chls A27, B22, A24, and A29 are all assigned as Chl *f* in the FRL-PSI structure from Kato *et al*.[2] but no nearby H-bond donors are present in their structural model. For A27, B22, and A24, the authors mention that the formyl groups appear to interact with O atoms of nearby Chl tails; however, this is chemically unlikely because both the formyl substituent and these keto-oxygen atoms exhibit (partial) negative charges that should repel one another. This is also the case with A29, where none of the nearby atoms on the His sidechain or the Chl tetrapyrrole it coordinates have H-bonding capacity. Because none of these Chls appear significantly π-stacked with a partner Chl, the generalizations derived from the LHC structural analysis (**Fig. 3**) do not support these Chls as being Chl *f*. We remind the reader that Chl *f*-binding sites are likely to be even more specific than those observed for Chl *b* sites in LHC proteins because Chl *f* must transfer energy uphill to Chl *a*, and thus the placement of Chl *f* molecules must avoid the creation of low-energy trapping sites. This suggests that situations where the Chl *f* formyl substituent exhibits no H-bonding in an experimentally derived molecular structure will be far less probable than the 21% of Chl *b* sites found in LHC that exhibit no obvious H-bonding partner.

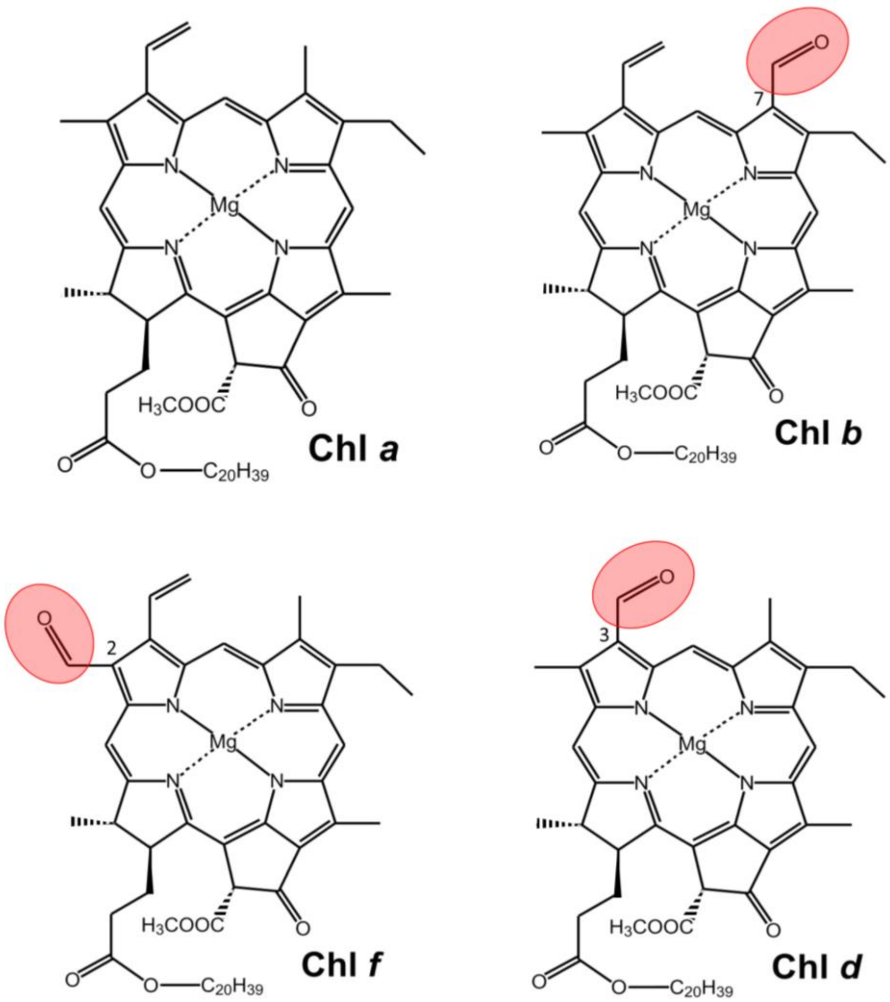

Supplementary Fig. 1 The four major Chls of cyanobacteria and higher plants[9]. The formyl groups that uniquely distinguish Chls *b*, *d*, and *f* are shaded. The esterifying alcohol is phytol ($C_{20}H_{39}O$).

```
PsaJ1 H. hongdechloris ---MDNLVKYLSTAPVVAAIWMAITAGVLIEFNRFFPDLLFHP------ 40
PsaJ1 F. thermalis     MDNQSPFFKFLSTAPVITTIWLFITAGILIEFNRFFPDLLFHPLP---- 45
PsaJ2 H. hongdechloris ---MSYFVKYLTSAPVMATLALVILSVVMIELNHIFPGLQYGYFHVAP- 45
PsaJ2 F. thermalis     -MEARYLFRYLSSAPVVATL--IIISVILIVLNYLFPGLQYGTFFHSLP 46
                             :.::*::***::::   * : ::* :* :**.* :
```

Supplementary Fig. 2 Sequence alignments supporting Chl B30 as Chl *f* in both *H. hongdechloris* and *F. thermalis* FRL-PSI. Sequence alignments were performed using Clustal Omega[10]. The conserved Tyr suggested to donate an H-bond to a Chl *f* formyl substituent in FRL-specific PsaJ2 is in a red box and the one well-resolved in the *F. thermalis* FRL-PSI density is bold. Clustal Omega similarity designator is shown below the sequences.

```
PsaA2 H. hongdechloris HANLAIHLVQFGTASLLVAHHMYAMPPYPYLATDYATVTSLFTHHVWI 404
PsaA2 F. thermalis     HAQLSINLAMLGSLSIIIAHHMYAMPPYPYLATDYGTVVSLFTHHVWI 407
                       **:*:*:*. :*: *:::*****************.**.*********
```

Supplementary Fig. 3 Sequence alignments supporting Chl A23 as a species-dependent Chl *f* site. The sequence alignment was performed using Clustal Omega[10]. Proposed H-bond donor is in bold and the red box shows the corresponding residue (and its Clustal Omega similarity designator) from the sequence where the residue is not conserved.

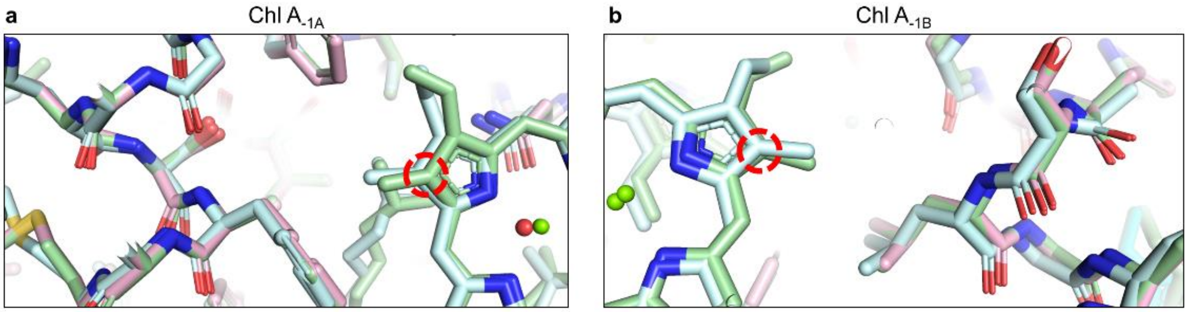

Supplementary Fig. 4 Environment of Chl A_{-1} sites. The structures from *H. hongdechloris* FRL-PSI, *F. thermalis* FRL-PSI, and a *C. thermalis* FRL-PSI homology model (polypeptides of PsaA2 and PsaB2 only, made using SwissModel[11] with templates of PsaA2 and PsaB2 from the *H. hongdechloris* FRL-PSI structure) are superimposed. A red dashed circle designates the C2 position where a formyl group would replace the methyl group if this Chl were to be Chl *f*.

Gisriel *et al.*[1]	Kato *et al.*[2]
B7	810B
B37	844A
A21	824A
B30	832B
A23	826A
A20	823A
A27	830A
B22	825B
A24	827A
A29	832A

Supplementary Table 1 Nomenclature conversion table for the FRL-PSI structure manuscripts. Kato *et al*.[2] note that the nomenclature adopted by their manuscript does not necessarily match that found in the associated Protein Data Bank[12,13] files.

Supplementary References

1. Gisriel, C. *et al.* The structure of Photosystem I acclimated to far-red light illuminates an ecologically important acclimation process in photosynthesis. *Sci. Adv.* **6**, eaay6415 (2020).
2. Kato, K. *et al.* Structural basis for the adaptation and function of chlorophyll *f* in photosystem I. *Nat. Commun.* **11**, 238 (2020).
3. Gan, F. *et al.* Extensive remodeling of a cyanobacterial photosynthetic apparatus in far-red light. *Science* **345**, 1312–1317 (2014).
4. Nürnberg, D. J. *et al.* Photochemistry beyond the red limit in chlorophyll *f*–containing photosystems. *Science* **360**, 1210–1213 (2018).
5. Cherepanov, D. A. *et al.* Evidence that chlorophyll *f* functions solely as an antenna pigment in far red-light Photosystem I from *Fischerella thermalis* PCC 7521. *Biochim. Biophys. Acta - Bioenerg.* **1861**, 148184. (2020).
6. Kaucikas, M., Nürnberg, D. J., Dorliac, G., Rutherford, A. W. & van Thor, J. J. Femtosecond visible transient absorption spectroscopy of chlorophyll *f*-containing Photosystem I. *Biophys. J.* **112**, 234–249 (2017).
7. Zamzam, N., Kaucikas, M., Nürnberg, D. J., Rutherford, A. W. & van Thor, J. J. Femtosecond infrared spectroscopy of chlorophyll *f*-containing photosystem I. *Phys. Chem. Chem. Phys.* **21**, 1224–1234 (2019).
8. Hastings, G. *et al.* Fourier transform visible and infrared difference spectroscopy for the study of P700 in photosystem I from *Fischerella thermalis* PCC 7521 cells grown under white light and far-red light: Evidence that the A_{-1} cofactor is chlorophyll *f*. *Biochim. Biophys. Acta - Bioenerg.* **1860**, 452–460 (2019).
9. Bryant, D. A., Hunter, C. N. & Warren, M. J. Biosynthesis of the modified tetrapyrroles—the pigments of life. *J. Biol. Chem.* **295**, 6888–6925 (2020).
10. Goujon, M. *et al.* A new bioinformatics analysis tools framework at EMBL-EBI. *Nucleic Acids Res.* **38**, W695-699 (2010).
11. Guex, N., Peitsch, M. C. & Schwede, T. Automated comparative protein structure modeling with SWISS-MODEL and Swiss-PdbViewer: A historical perspective. *Electrophoresis* **30**, Suppl 1, S162-173 (2009).
12. Berman, H. M. *et al.* The Protein Data Bank. *Nucl. Acids Res.* **28**, 235–242 (2000).
13. Berman, H. M., Henrick, K. & Nakamura, H. Announcing the worldwide Protein Data Bank. *Nat. Struct. Mol. Biol.* **10**, 980 (2003).
